# Alterations in serum protein glycopatterns related to small cell lung cancer, adenocarcinoma and squamous carcinoma of the lung†

**DOI:** 10.1039/c9ra10077f

**Published:** 2020-02-18

**Authors:** Liyuan Jia, Tianran Ma, Yiqian Liang, Haoqi Du, Jian Shu, Xiawei Liu, Zhiwei Zhang, Hanjie Yu, Mingwei Chen, Zheng Li

**Affiliations:** Laboratory for Functional Glycomics, College of Life Sciences, Northwest University Xi'an 710069 P. R. China zhengli@nwu.edu.cn; Department of Respiratory and Critical Care Medicine, The First Affiliated Hospital of School of Medicine of Xi'an, Jiaotong University Xi'an 710061 China

## Abstract

*Background*: The main reason why lung cancer has maintained a high rate of morbidity and mortality is that its early diagnosis is difficult. No current lung cancer screening is recommended by any major medical organization due to the lack of sensitive and specific screening technologies. Thus, this study aimed to systematically investigate the correlation between the alterations in serum glycosylation and three main types of lung cancers (SCLC, ADC and SqCC). *Materials and methods*: We investigated the protein glycopatterns in sera from 333 subjects (65 healthy volunteers, 38 benign lung disease patients, 49 small cell lung cancer patients, and 181 NSCLC patients) using a lectin microarray. A serum microarray was produced to evaluate and verify the terminal carbohydrate moieties of the glycoproteins in individual serum samples from 30 cases simultaneously. *Results*: There were 16 lectins (*e.g.*, RCA120, BS-I, and UEA-I), 24 lectins (*e.g.*, HHL, PTL-I, and MAL-II), and 18 lectins (*e.g.*, GSL-I, LEL, and ACA) that exhibited significant differences in serum protein glycopatterns in the patients with SCLC, ADC and SqCC compared with the controls (HV and BPD). There were 6 lectins (*e.g.*, EEL, NPA, and LEL) that exhibited significantly increased NFIs in ADC and SqCC compared with SCLC. Also, there were 5 lectins (*e.g.*, Jacalin, BS-I, and UEA-I) that exhibited significantly decreased NFIs in ADC compared with SCLC and SqCC. *Conclusions*: This study can facilitate the discovery of potential biomarkers for the differential diagnosis of lung cancer based on the precise alteration in serum protein glycopatterns.

## Abstract 2

The main reason why lung cancer has maintained a high rate of morbidity and mortality is that its early diagnosis is difficult.

## Graphical abstract

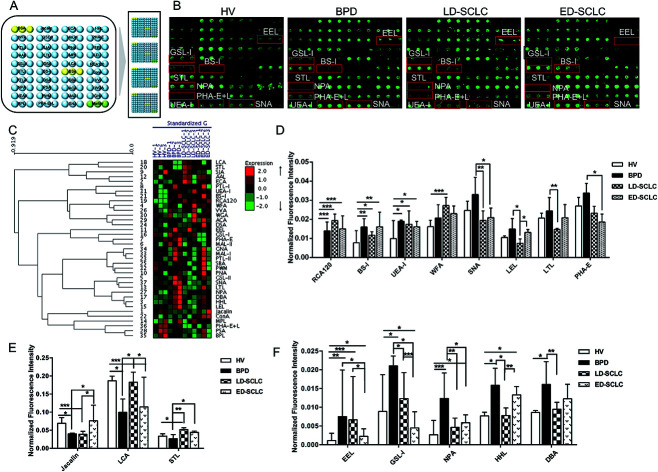

## 1 Introduction

Lung cancer is the most common malignant tumor and the first leading cause of cancer-related deaths among men and the second leading cause of cancer-related deaths among women worldwide.^1–4^ The main reason why lung cancer has maintained a high rate of morbidity and mortality is that its early diagnosis is difficult. Most lung cancer patients have metastatic primary lung cancer at the time of diagnosis, and their five-year survival rate is only 3.5%. Therefore, the low survival rate of lung cancer is closely related to the abovementioned reasons.^3,5^ In contrast, early-stage lung cancer has relatively favorable survival.^6^ However, currently, there is no lung cancer screening recommended by any major medical organization due to the lack of sensitive and specific screening technologies.^7^ Lung cancers are broadly classified into two types, namely, small cell lung cancers (SCLC) and non-small cell lung cancers (NSCLC), which is based upon the microscopic appearance of the tumor cells. SCLC comprises about 10–15% of lung cancers, which metastasize rapidly to many sites within the body and are most often discovered after they have spread extensively. The clinical stage of SCLC can be divided into limited diseases and extensive diseases. However, NSCLC is the most common lung cancer, accounting for about 85% of all cases, which has several main types: squamous cell lung cancer (SqCC), lung adenocarcinoma (ADC), large cell carcinoma (LCC) and lung adenosquamous carcinoma (ASC). ADC forms the largest group (approximately 60%) among all NSCLC and is thereby the most frequent cause of tumor-related deaths.^8–10^

Tissues and blood were used extensively in early attempts to detect lung cancer. However, it is difficult to acquire enough tissue samples for molecular marker detection because most lung cancer patients are often diagnosed late, and they are not suitable for surgery. In contrast, serum samples are easy to obtain *via* minimally invasive methods, and they are suitable and convenient for molecular marker detection. The serum isocitrate dehydrogenase 2 (IDH2) can be a new and reliable biomarker for the diagnosis and prognosis of NSCLC.^11^ Currently, some tumor markers are used to detect lung cancer, such as carcinoembryonic antigen (CEA), serum cytokeratin 19 fragment (Cyfra 21-1) and progesterone-releasing peptide (pro-GRP). However, the relatively low sensitivity and specificity of these markers in the detection of cancer cells make their application unsatisfactory in the early diagnosis and monitoring of diseases.^12,13^ Furthermore, some target prodrugs have been designed and synthesized for the precision therapy of NSCLC. The prodrug polyamine analog gefitinib (PPG) offers reliable and rapid data for the diagnosis of NSCLC within 4 h.^14,15^

There is significant correlation between aberrant glycosylation modification and many diseases. Many types of genetic diseases are result of congenital disorder of glycosylation. Alterations in glycan structures are closely related to the mechanism of numerous diseases, suggesting that a change in these glycans may play a critical role in tumor formation and development.^16^ For example, most of the proteins in the serum are secreted by the liver, thus many studies have identified *N*-glycan alterations in the development of liver disease, which is the basis of many investigations. Alterations in core fucosylation, outer-arm fucosylation, sialylation and glycan branching have been found in the serum of patients with liver disease.^17^ Similarly, there are also many groups that give greater insight into the underlying biological mechanisms of aberrant glycosylation in lung cancer.^18,19^ For example, the role of α-1,3-fucosyltransferase (Fut3) in the synthesis of sLex and prognosis of its physiological function have been assessed in lung cancer. The accumulation of tumor-associated antigens, such as sLex, was mostly studied in the sera of patients with lung cancer. The level of core fucosylation increased in ADC compared with nonmalignant tissue. However, although the pathogenic mechanism is not completely clear, glycosylation modification is closely related to many diseases. Changes in the glycan structures of serum proteins are an indication when a disease is present, which can provide important information in the pathogenesis and progression for various lung diseases.^20^ The differential glycosylation patterns of lung tumor tissue and nonmalignant tissue at the level of individual glycan structures were examined using nLC-chip-TOF-MS.^21^ Also, a serum mass profile-based signature identifying patients with early lung cancer was developed,^22,23^ which profiled the low-molecular-weight serum fraction by MALDI-MS and revealed several components with abundances discriminating patients with early lung cancer from healthy high-risk smokers.^24^ Additionally, 38 glycopeptides from 22 different proteins were significantly differentially abundant in the serum of lung cancer using LC-MS/MS, and the differential expression levels of serum proteins harboring selective glycopeptide candidates were confirmed using sandwich-based ELISAs.^25^ Lectins are also carbohydrate–binding proteins that can distinguish glycans based on structural differences.^26^ Researchers were able to distinguish between patients with ADC, SQLC and SqCC and patients with benign conditions using lectin arrays to detect the A1AT-specific glycosylation changes in individual serum glycoproteins.^27^ The lectin microarray can quickly detect many different types of glycosylation forms simultaneously. Thus, the emergence of this high-throughput sugar group technology has become the main method to study the glycosylation of various protein samples.^28^

SCLC, ADC and SqCC are the most common lung cancers in clinic. LCC and ASC are unusual types, which account for less than 7% of lung cancers. In this study, we aimed to systematically investigate the correlation between the alterations in serum glycosylation and the three main types of lung cancers (SCLC, ADC and SqCC) using lectin microarrays, provide new basic insight into serum protein glycopatterns, and compare different or similar alterations in glycoprotein glycopatterns between SCLC, ADC and SqCC.

## 2 Materials and methods

### 2.1 Study approval

The collection and use of all human serum for research presented here were approved by the Ethical Committee of Northwest University and the First Hospital of Medicine College of Xi'an Jiaotong University (Xi'an, China). Written informed consent was received from participants for the collection of their whole serum. This study was conducted in accordance with the Ethical Guidelines of the Declaration of Helsinki.

### 2.2 Serum collection

Serum samples were collected from 65 healthy volunteers (median age 69.7 years [range 60.5–84.5], 60% male), and 268 patients with lung diseases (66.7% male). These patients had two main types, SCLC and NSCLC. SCLC patients (*n* = 87) including (1) benign pulmonary disease (BPD, *n* = 38, median age 60.1 years [range 42.0–83.0]), (2) limited disease small cell lung cancer (LD-SCLC, *n* = 23, median age 69 years [range 65.5–76.0]), and (3) extensive disease small cell lung cancer (ED-SCLC, *n* = 26, median age 59.5 years [range 46.5–73.5]). The NSCLC patients (*n* = 181) included: (1) lung adenocarcinoma early stage (ADC-ES, *n* = 17, median age 59.0 years [range 41.5–71.0]), (2) lung adenocarcinoma advanced stage (ADC-AS, *n* = 73, median age 56.0 years [range 45.5–63.5]), (3) squamous cell lung cancer early stage (SqCC-ES, *n* = 21, median age 45.0 years [range 37.0–58.5]), and (4) squamous cell lung cancer advanced stage (SqCC-AS, *n* = 70, median age 62.5 years [range 52.0–72.5]). The baseline characteristics of HVs and patients with lung diseases are summarized in Table 1.

**Table 1:** Baseline characteristics of healthy volunteers and patients of benign lung diseases and lung cancer-induced small cell lung cancer, adenocarcinoma and squamous carcinoma

Subject group	Healthy volunteers	BPD	SCLC		ADC					SqCC				
			LS	ES	I	II	IIIa	IIIb	IV	I	II	IIIa	IIIb	IV
*n*	65	38	23	26	7	10	20	25	28	10	11	15	25	30
Males, (*n*)%	35 (67.7)	11 (55.8)	16 (69.6)	16 (69.7)	2 (28.6)	5 (50)	8 (40)	7 (28)	9 (32.1)	5 (50)	10 (91.0)	13 (86.7)	17 (11.3)	24 (80)
Age, years	69.7 (60.5–84.5)	60.1 (42.0–83.0)	69 (65.5–76.0)	59.5 (46.5–73.5)	59.6 (44–77)	59.2 (40–77)	62.3 (42–74)	53.4 (41–68)	59.8 (41–74)	56 (39–72)	65.1 (44–82)	52 (39–72)	65.1 (44–82)	62.85 (40–65)
Pack/year, mean (±SD)			35.91 (±26.05)	38.24 (±30.45)	2.75 (±2.76)	16.43 (±20.85	28.93 (±27.29)	36.48 (±25.94)	29.18 (±28.17)	35.92 (±23.24)	40.06 (±35.58)	31.45 (±22.21)	36.48 (±25.94)	29.18 (±28.17)
Current smoker, *N* (%)			2 (8.70%)	1 (3.85%)	0 (0%)	0 (0%)	0 (0%)	0 (0%)	0 (0%)	0 (0%)	1 (9.10%)	2 (13.3%)	8 (32%)	6 (20%)
Total serum protein (g L^−1^)	63.22 ± 3.87	61.58 ± 1.16	58.42 ± 3.53	63.98 ± 2.15	64.01 ± 1.25	70.18 ± 1.19	58.82 ± 2.64	66.9 ± 3.04	57.19 ± 3.01	62.85 ± 2.53	68.17 ± 1.85	58.24 ± 2.34	63.92 ± 1.53	66.24 ± 2.43

Serum was separated immediately *via* centrifugation at 13 000 × *g* for 10 min at 4 °C. An EDTA-free inhibitor cocktail (Halt protease inhibitor; Thermo Scientific Pierce Protein Research Products, Rockford, IL, USA) was added immediately at a concentration of 10 μL mL^−1^ serum. The produced serum was aliquoted into small portions, immediately frozen on dry ice and stored at −80 °C. The protein concentration was determined using the BCA assay.

### 2.3 Lectin microarrays

To normalize the differences between subjects and to tolerate individual variation, the serum samples from HV and patients with SCLC, ADC and SqCC were pooled. Total proteins in the sera were labeled using Cy3 fluorescent dye and purified using a Sephadex-G25 column. Lectin microarrays were utilized as previously described.^29,30^ Briefly, 37 lectins with different binding preferences covering N- and O-linked glycans were spotted on homemade epoxysilane-coated slides. Each lectin was spotted in triplicate per block, with quadruplicate blocks on one slide. After immobilization, the slides were blocked with blocking buffer containing 2% BSA in 1 × PBS (0.01 M phosphate buffer containing 0.15 M NaCl, pH 7.4) for 1 h and rinsed twice with 1 × PBS. Then the blocked slide was incubated with Cy3-labeled salivary proteins diluted in 0.6 mL of incubation buffer for 3 h at room temperature. After incubation, the microarray was rinsed twice with 1 × PBST (0.2% Tween 20 in 1 × PBS) for 5 min each, and finally rinsed in 1 × PBS before drying. The microarrays were scanned using a Genepix 4000B confocal scanner (Axon Instruments, Foster City, Calif., USA) set at 70% photomultiplier tube and 100% laser power. The acquired images were analyzed at 532 nm for Cy3 detection using the Genepix 6.0 software.

### 2.4 Data acquisition and analysis

The acquired images were analyzed at 532 nm for Cy3 detection using the Genepix 3.0 software. The average background was subtracted, and values less than the average background ± 1.5 standard deviations (SD) were removed from each data point. The median of the effective data points for each lectin was globally normalized to the sum of medians of all the effective data points for each lectin in a block. Each pooled sample was observed consistently by three repeated slides and the normalized medians of each lectin from 9 repeated blocks were averaged and its SD was counted. The normalized data of the parallel groups were compared with each other based on fold change, according to the following criteria: fold change ≥ 1.5 or ≤0.67 in pairs indicated up-regulation or down-regulation, respectively. Differences between two arbitrary data sets or multiple data sets were tested by Student's *t* test or one-way ANOVA for each lectin signal using SPSS statistics. The original data was further analyzed using Expander 6.0 (http://acgt.cs.tau.ac.il/expander/) to perform hierarchical clustering analysis.^31–33^

### 2.5 Serum microarrays

A sera microarray was produced by 120 individual serum samples from HVs (*n* = 30) and patients with BPD (*n* = 30), SCLC (*n* = 30), ADC (*n* = 30) and SqCC (*n* = 30) according to our previous protocols.^30,34^ These serum samples were randomly chosen from the 333 samples we collected before. The baseline characteristics of HVs and patients with lung diseases are summarized in Table 1. Briefly, the serum samples were dissolved in spotting buffer containing 0.5 mg mL^−1^ BSA in 1 × PBS, pH 7.4, to a concentration of 1 mg mL^−1^ before spotting on homemade epoxysilane-coated slides with Stealth micro spotting pins (SMP-10B). After immobilization, the slides were blocked with the blocking and incubation buffers (ESI Appendix†) for 1 h and rinsed twice with 1 × PBS. Cy3-labeled lectin diluted in 0.5 mL of buffer (ESI Table 7†) was incubated on the blocked slide for 3 h at room temperature in the dark. The slide was washed and dried *via* centrifugation at 600 rpm for 5 min. The slide was scanned using a Genepix 4000B confocal scanner at 70% photomultiplier tube and 100% laser power settings, and the acquired images were analyzed at 532 nm for Cy3 detection. All values less than average background ± 2SD were removed from each data point, and the median of the effective data points of each sample was counted. The medians from one group were averaged and expressed as the mean ± SD. Differences between two arbitrary groups or multiple groups of medians were tested using Student's *t*-test or one-way ANOVA for each serum sample using SPSS version 19.

## 3 Results

### 3.1 Alterations of serum glycopatterns from patients with SCLC

**Fig. 1:**
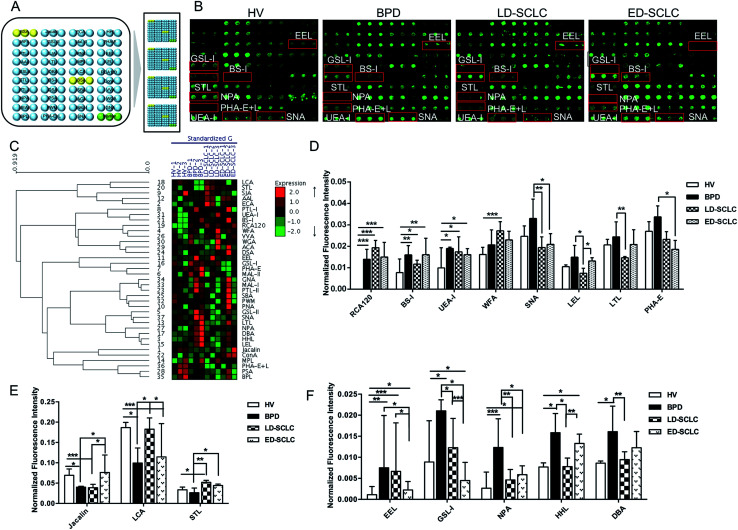
Glycopatterns of serum glycoproteins from male and female HVs and lung disease patients with BPD, LD-SCLC and ED-SCLC using a lectin microarray. (A) Layout of the lectin microarray. Each lectin was spotted in triplicate per block, with triplicate blocks on one slide. Cy3-labeled BSA was spotted as a location marker and BSA as a negative control. (B) Profiles of Cy3-labeled serum proteins from HV and lung disease patients with BPD, LD-SCLC and ED-SCLC bound to the lectin microarrays. A portion of the slide with three replicates of the lectin arrays is shown. The lectin microarrays revealed a significant difference, as marked with red frames. (C) Heat map and hierarchical clustering analysis of the 37 lectins with three biological replicates. The samples are listed in the columns, and the lectins are listed in the rows. The color and intensity of each square indicate the expression levels relative to the other data in the row. Red, high; green, low; and black, medium. (D) Three lectins revealed significant differences between HV and all groups of patients with SCLC, and five lectins revealed significant differences between BPD and SCLC patients, lectins showing decreasing trends of NFIs (*p* < 0.05). (E) Three lectins showing a decreasing trend of NFIs (fold change ≤ 0.67, *p* < 0.05) in parts of groups with SCLC compared with HV and BPD according to one-way ANOVA (*p < 0.05, **p < 0.01, and ***p < 0.001). (F) Five lectins showing increasing trend of NFIs (fold change ≥ 1.5, *p* < 0.05) in some of the groups with SCLC compared with HV and BPD according to one-way ANOVA (*p < 0.05, **p < 0.01, and ***p < 0.001).

The subjects were chosen at the same or similar age stage and the proportionality subjects in each group was similar to avoid differences between subjects and tolerate individual variations. The layout of the lectin microarrays and glycopatterns of the Cy3-labeled pooled serum proteins from HV, BPD, LD-SCLC, and ED-SCLC bound to the lectin microarrays are shown in Fig. 1A and B. The normalized fluorescent intensities (NFIs) for each lectin are summarized as the mean value ± SD in ESI Table 1.† Specifically, (1) results showing a significant increase in NFIs (fold change ≥ 1.50, *p* < 0.05), (2) results showing a significant decrease in NFIs (fold change ≤ 0.67, *p* < 0.05), and (3) results showing an almost even level in NFIs (fold change range from 0.67 to 1.50, no significant difference). All the results based on fold change in pairs (with *p*-values lower than 0.05) with the NFIs of each lectin from HV, BPD, LD-SCLC, and ED-SCLC are shown in ESI Table 2.† The results showed that there were 16 lectins giving a significantly differential signal in pooled serum from HV, BPD, LD-SCLC, and ED-SCLC. The generated data from three biological replicates were imported into EXPANDER 6.0 to perform a hierarchical clustering analysis (Fig. 1C).

The results showed that the β-Gal and Galβ-1 binder RCA120, the α-Gal and α-GalNAc binder BS-I, and the Fucα1-2Galβ1-4Glc(NAc) binder UEA-I exhibited significantly increased NFIs in BPD, LD-SCLC, and ED-SCLC compared with HV (all fold change ≥ 1.51, *p* ≤ 0.037); however, there was no significant difference between BPD, LD-SCLC, and ED-SCLC. The terminal GalNAcα/β1-3/6Gal binder WFA had significantly increased NFIs in LD-SCLC compared with HV (fold change = 1.69, *p* < 0.001). Also, the Siaα2-6Gal/GalNAc binder SNA, (GlcNAc)_*n*_ and high mannose-type *N*-glycans binder LEL and the Fucα1-2Galβ1-4GlcNAc binder LTL were associated with decreased NFIs in LD-SCLC compared with BPD (all fold change ≤ 0.60, *p* ≤ 0.021); however, SNA showed significantly decreased NFIs in ED-SCLC compared with BPD (fold change = 0.55, *p* = 0.016), and an increase in the NFIs of LEL was observed in ED-SCLC compared with LD-SCLC (fold change = 1.76, *p* = 0.014). The bisecting GlcNAc binder PHA-E was associated with decreased NFIs in ED-SCLC compared with BPD (fold change = 0.63, *p* = 0.049) (Fig. 1D).

The Galβ1-3GalNAcα-Ser/Thr (T) binder Jacalin had significantly decreased NFIs in BPD and LD-SCLC compared with HV (all fold change ≤ 0.58, *p* ≤ 0.043), and significantly increased NFIs in ED-SCLC compared with BPD and LD-SCLC (all fold change ≥ 1.91, *p* ≤ 0.042). The α-d-Man and Fucα-1,6GlcNAc binder LCA had a decrease in NFIs in BPD and ED-SCLC compared with HV (all fold change ≤ 0.63, *p* ≤ 0.028), but an increase in NFIs was observed in LD-SCLC compared with BPD and ED-SCLC (all fold change ≥ 1.84, *p* ≤ 0.032). The trimers and tetramers of the GlcNAc binder STL had significantly increased NFIs in LD-SCLC and ED-SCLC compared with BPD, and had significantly increased NFIs in LD-SCLC compared with HV (all fold change ≥ 1.55, *p* ≤ 0.046) (Fig. 1E).

The Galα1-3(Fucα1-2) Gal binder EEL showed an increase in NFIs in BPD, LD-SCLC and ED-SCLC compared with HV (all fold change ≥ 1.99, *p* ≤ 0.23), but a decrease in NFIs was observed in ED-SCLC compared with BPD and LD-SCLC (all fold change ≤ 0.34, *p* ≤ 0.047). Also, the αGalNAc/αGal binder GSL-I was associated with decreased NFIs in LD-SCLC and ED-SCLC compared with BPD (all fold change ≤ 0.58, *p* ≤ 0.039) and a decrease in NFIs was observed in ED-SCLC compared with HV and LD-SCLC (all fold change ≤ 0.58, *p* ≤ 0.039). The high-mannose binder NPA, high-mannose binder HHL, and αGalNAc and GalNAcα1-3(Fucα1-2) Gal binder DBA showed a significant increase in NFIs in BPD compared with HV and LD-SCLC (all fold change ≥ 1.87, *p* ≤ 0.010) (Fig. 1F).

### 3.2 Alterations of serum glycopatterns from patients with ADC

**Fig. 2:**
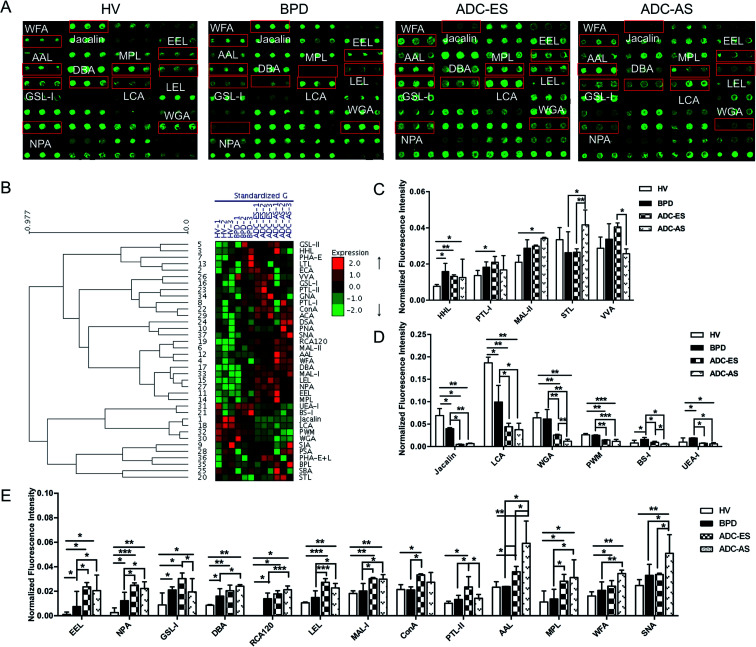
Glycopatterns of the serum glycoproteins from male and female HVs and lung disease patients with BPD, ADC-ES and ADC-AS using a lectin microarray. (A) Layout of the lectin microarray. Each lectin was spotted in triplicate per block, with triplicate blocks on one slide. Cy3-labeled BSA was spotted as a location marker and BSA as a negative control. The profiles of Cy3-labeled serum proteins from HV and lung disease patients with BPD, ADC-ES and ADC-AS bound to the lectin microarrays. A portion of the slide with three replicates of the lectin arrays is shown. The lectin microarrays revealed a significant difference, as marked with red frames. (B) Heat map and hierarchical clustering analysis of the 37 lectins with three biological replicates. The samples are listed in the columns, and the lectins are listed in the rows. The color and intensity of each square indicate the expression levels relative to the other data in the row. Red, high; green, low; and black, medium. (C) Three lectins revealed significant differences between the HV and ADC patients, and two lectins revealed significant differences between the BPD and ADC patients, with the lectins showing decreasing trends of NFIs (*p* < 0.05). (D) Six lectins showed a decreasing trend of NFIs (fold change ≤ 0.67, *p* < 0.05) in parts of groups with ADC compared with HV and BPD according to one-way ANOVA (*p < 0.05, **p < 0.01, and ***p < 0.001). (E) Eleven lectins showed increasing trend of NFIs (fold change ≥ 1.5, *p* < 0.05) in some of the groups with ADC compared with HV and BPD according to one-way ANOVA (*p < 0.05, **p < 0.01, and ***p < 0.001).

The glycopatterns of the Cy3-labeled pooled serum proteins from HV, BPD, ADC-ES, and ADC-AS bound to the lectin microarrays are shown in Fig. 2A. The NFIs for each lectin are summarized as the mean values ± SD in ESI Table 3.† All the results based on fold change in pairs (with *p*-values lower than 0.05) with the NFIs of each lectin from HV, BPD, ADC-ES, and ADC-AS are shown in ESI Table 4.† The results showed that there were 24 lectins showing a significantly differential signal in the pooled serum from HV, BPD, ADC-ES, and ADC-AS. The generated data from three biological replicates was imported into EXPANDER 6.0 to perform hierarchical clustering analysis (Fig. 2B). The results showed that HHL exhibited significantly increased NFIs in all the patients compared with HV (all fold change ≥ 1.64, *p* ≤ 0.041). The GalNAc binder PTL-I had significantly increased NFIs in ADC-ES compared with HV (fold change = 1.55, *p* = 0.016); however, the Siaα2-3Galβ1-4Glc(NAc)/Glc binder MAL-II exhibited increased NFIs in ADC-AS compared with HV (fold change = 0.64, *p* < 0.001). STL was associated with increased NFIs in ADC-AS compared with BPD and ADC-ES (fold change = 1.60 and 1.57, *p* = 0.017 and 0.009). The terminal GalNAc binder VVA exhibited significantly decreased NFIs in ADC-AS compared with ADC-ES (fold change = 0.64, *p* = 0.011) (Fig. 2C).

Both Jacalin and LCA exhibited significantly decreased NFIs in all the patients compared with HV (all fold change ≤ 0.58, *p* ≤ 0.043) and in ADC-ES and ADC-AS compared with BPD (all fold change ≤ 0.45, *p* ≤ 0.032). The multivalent Sia and (GlcNAc)_*n*_ binder WGA and the (LacNAc)_*n*_ binder PWM exhibited significantly decreased NFIs in ADC-ES and ADC-AS compared with HV and BPD (all fold change ≤ 0.59, *p* ≤ 0.008), and WGA showed a significant decreasing trend from ADC-ES to ADC-AS (fold change = 0.46, *p* = 0.020). BS-I and UEA-I exhibited significantly increased NFIs in BPD compared with HV (fold change = 2.07 and 1.91, *p* = 0.043 and 0.045), and decreased NFIs in ADC-ES and ADC-AS compared with BPD (all fold change ≤ 0.59, *p* ≤ 0.030), and BS-I also showed a significant decreasing trend from BPD and ADC-ES to ADC-AS (all fold change ≤ 0.56, *p* ≤ 0.038), and UEA-I exhibited a significant decrease in ADC-AS compared with HV (fold change = 0.60, *p* = 0.012) (Fig. 2D).

EEL, NPA, GSL-I, DBA, and RCA120 were associated with significantly increased NFIs in all the patients compared with HV (all fold change ≥ 1.87, *p* ≤ 0.049), and the NFIs of EEL and NPA in ADC-ES and ADC-AS were significantly increased compared with BPD (all fold change ≥ 1.82, *p* ≤ 0.040). The NFIs of DBA and RCA120 showed a significant increasing trend from BPD and ADC-ES to ADC-AS (all fold change ≥ 1.52, *p* ≤ 0.033). GSL-I, LEL, the Galβ-1/4GlcNAc/Siaα2-3Gal binder MAL-I, the high-mannose binder ConA, and the Gal binder PTL-II exhibited significantly increased NFIs in ADC-ES compared with HV and BPD (all fold change ≥ 1.50, *p* ≤ 0.049), and decreased NFIs in ADC-AS compared with ADC-ES (all fold change ≤ 0.64, *p* ≤ 0.022). The Fucα1-6 GlcNAc (core fucose) binder AAL, the Galβ1-3GalNAc/GalNAc binder MPL, the terminating in GalNAcα/β1-3/6Gal binder WFA, and SNA showed significantly increased NFIs in ADC-AS compared with HV, BPD and ADC-ES (all fold change ≥ 1.51, *p* ≤ 0.015), except for the NFIs of MPL, which were not significantly different between ADC-ES and ADC-AS, and the NFIs of WFA and SNA were not significantly different between ADC-ES and BPD (Fig. 2E).

### 3.3 Alterations of serum glycopatterns from patients with SqCC

**Fig. 3:**
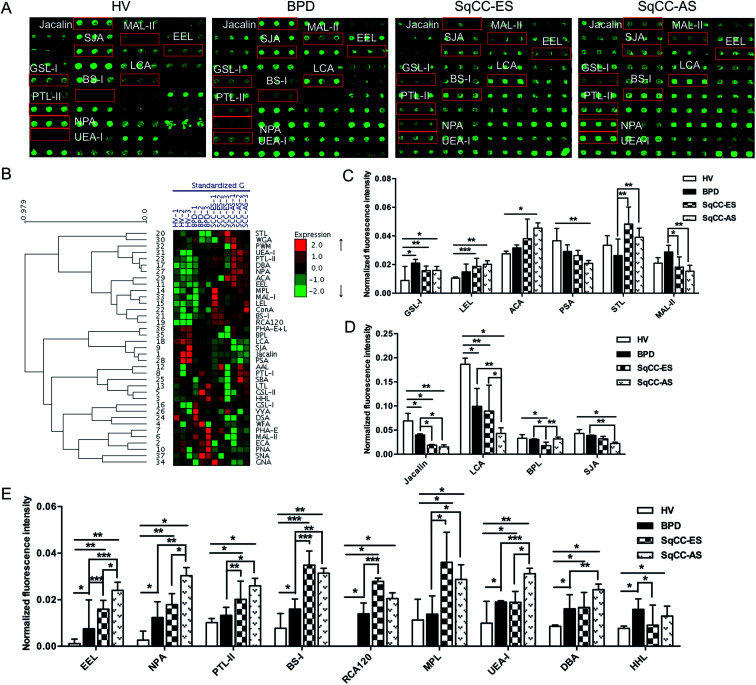
Glycopatterns of the serum glycoproteins from male and female HVs and lung disease patients with BPD, SqCC-ES and SqCC-AS using a lectin microarray. (A) Layout of the lectin microarray. Each lectin was spotted in triplicate per block, with triplicate blocks on one slide. Cy3-labeled BSA was spotted as a location marker and BSA as a negative control. The profiles of Cy3-labeled serum proteins from HV and lung disease patients with BPD, SqCC-ES and SqCC-AS bound to the lectin microarrays. A portion of the slide with three replicates of the lectin arrays is shown. The lectin microarrays revealed significant differences, as marked with red frames. (B) Heat map and hierarchical clustering analysis of the 37 lectins with three biological replicates. The samples are listed in the columns, and the lectins are listed in the rows. The color and intensity of each square indicate the expression levels relative to the other data in the row. Red, high; green, low; and black, medium. (C) Four lectins revealed significant differences between the HV and SqCC patients, and two lectins revealed significant differences between the BPD and SqCC patients, with the lectins showing decreasing trends of NFIs (*p* < 0.05). (D) Four lectins showed decreasing trend of NFIs (fold change ≤0.67, *p* < 0.05) in some of the groups with SqCC compared with HV and BPD according to one-way ANOVA (*p < 0.05, **p < 0.01, and ***p < 0.001). (E) Nine lectins showed an increasing trend of NFIs (fold change ≥ 1.5, *p* < 0.05) in some of the groups with SqCC compared with HV and BPD according to one-way ANOVA (*p < 0.05, **p < 0.01, and ***p < 0.001).

The glycopatterns of the Cy3-labeled pooled serum proteins from HV, BPD, SqCC-ES, and SqCC-AS bound to the lectin microarrays are shown in Fig. 3A. The NFIs for each lectin are summarized as the mean value ± SD in ESI Table 5.† All the results based on fold change in pairs (with *p*-values lower than 0.05) with the NFIs of each lectin from the HV, BPD, SqCC-ES, and SqCC-AS groups are shown in ESI Table 6.† The results showed that there were 18 lectins to give a significantly differential signal in the pooled serum from HV, BPD, SqCC-ES, and SqCC-AS. The generated data from three biological replicates were imported into EXPANDER 6.0 to perform hierarchical clustering analysis (Fig. 3B). The results showed that both GSL-I and LEL significantly increased the NFIs in the patients compared with HV (all fold change ≥ 1.80, *p* ≤ 0.037), except for the NFIs of LEL had no difference between BPD and HV. The Galβ1-3GalNAcα-Ser/Thr (T) binder ACA had significantly increased NFIs in SqCC-AS compared with HV (fold change = 1.68, *p* = 0.004) and the α-d-Man binder PSA had significantly decreased NFIs in SqCC-AS compared with HV (fold change = 0.58, *p* = 0.019). STL exhibited significantly increased NFIs in SqCC-ES and SqCC-AS compared with BPD (fold change = 1.85 and 1.51, *p* = 0.006 and 0.002). MAL-II exhibited significantly decreased NFIs in the SqCC-ES and SqCC-AS groups compared with BPD (fold change = 0.64 and 0.54, *p* = 0.011 and 0.008) (Fig. 3C).

Both Jacalin and LCA exhibited significantly decreased NFIs in the patients compared with HV (all fold change ≤ 0.58, *p* ≤ 0.043), and the NFIs of Jacalin in SqCC-ES and SqCC-AS were significantly lower than that of BPD (all fold change ≤ 0.48, *p* ≤ 0.026), and the NFIs of LCA in SqCC-AS were significantly lower than that of BPD and SqCC-ES (fold change = 0.44 and 0.48, *p* = 0.008 and 0.013). The Galβ1-3GalNAc binder BPL exhibited significantly decreased NFIs in SqCC-ES compared with HV and BPD (fold change = 0.54 and 0.58, *p* = 0.038 and 0.015), but an increase in the NFIs was observed in SqCC-AS compared with SqCC-ES (fold change = 1.79, *p* = 0.008). The terminal in GalNAc and Gal binder SJA exhibited significantly decreased NFIs in SqCC-AS compared with HV and BPD (fold change = 0.52 and 0.58, *p* = 0.012 and 0.003) (Fig. 3D).

The NFIs of EEL, NPA, and PTL-II exhibited a significant increasing trend in HV, BPD, and SqCC-ES to SqCC-AS (all fold change ≥ 1.52, *p* ≤ 0.030); however, BS-I, RCA120, and MPL exhibited significantly increased NFIs in SqCC-ES and SqCC-AS compared with HV and BPD (all fold change ≥ 2.00, *p* ≤ 0.028). UEA-I and DBA exhibited significantly increased NFIs in SqCC-AS compared with HV, BPD, and SqCC-ES (all fold change ≥ 1.87, *p* ≤ 0.047) and their NFIs in BPD and SqCC-ES were significantly higher than that of HV (all fold change ≥ 1.54, *p* ≤ 0.006). The high-mannose binder HHL exhibited significantly increased NFIs in BPD and SqCC-AS compared with HV (fold change = 2.06 and 1.71, *p* = 0.017 and 0.015), but a decrease in the NFIs was observed in SqCC-ES compared with the BPD (fold change = 0.58, *p* = 0.015) (Fig. 3E).

### 3.4 Individual validation of the different serum glycopatterns in patients with lung cancer

**Fig. 4:**
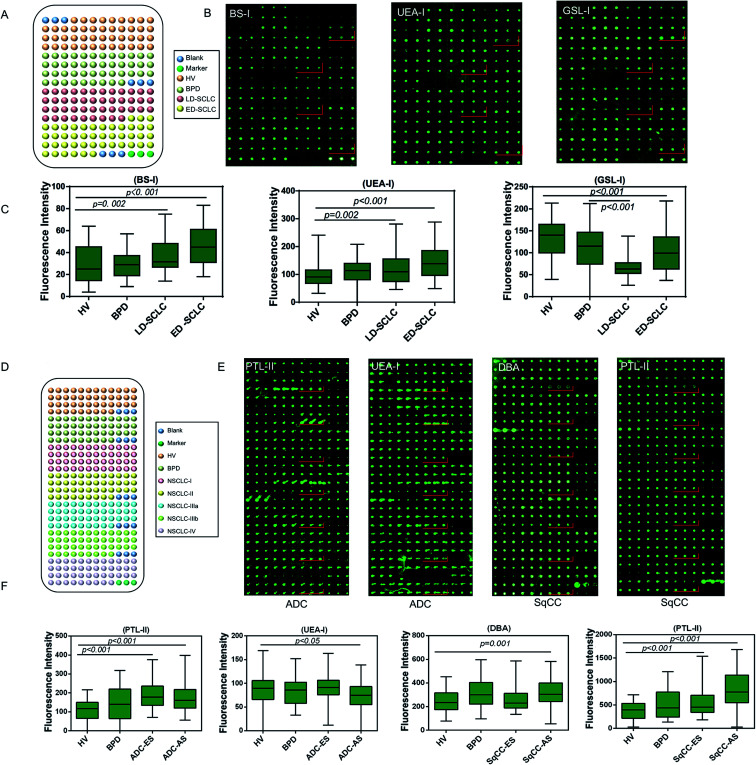
Validation of the differential expressions of the glycopatterns in the serum associated with SCLC, ADC and SqCC. (A) Layout of the serum microarrays. A total of 120 serum samples ranging from HV (*n* = 30) and from patients with BPD (*n* = 30), LD-SCLC (*n* = 30), and ED-SCLC (*n* = 30) were dissolved in spotting buffer to a concentration of 1 mg mL^−1^ and spotted on epoxysilane-coated slides. Each sample was spotted in triplicate per block, with two continuous blocks on one slide. (B) Scan images of Cy3-labeled lectins binding to a serum microarray. Here, a block of the slide with two sequential blocks is shown. (C) Box plot analysis of the original data obtained from the serum microarrays. Error bars represent 95% confidence intervals for the means. The statistical significance of the differences between groups is indicated by the *p*-value. (D) Layout of the serum microarrays. A total of 210 serum samples ranging from HV (*n* = 30) and from patients with BPD (*n* = 30), ADC/SqCC-I (*n* = 30), ADC-II/SqCC (*n* = 30), ADC-IIIa/SqCC (*n* = 30), ADC-IIIb (*n* = 30) and ADC/SqCC-IV (*n* = 30) were dissolved in spotting buffer to a concentration of 1 mg mL^−1^ and spotted on epoxysilane-coated slides. Each sample was spotted in triplicate per block, with two continuous blocks on one slide. (E) Scan images of Cy3-labeled lectins binding to a serum microarray. Here, a block of the slide with two sequential blocks is shown. (F) Box plot analysis of the original data obtained from the serum microarrays. Error bars represent 95% confidence intervals for the means. The statistical significance of the differences between groups was indicated by the *p*-value.

A serum microarray was fabricated to validate the differential expression levels of serum glycopatterns among HV, BPD, LD-SCLC, and ED-SCLC by spotting 120 individual serum samples (20 male and 10 female samples randomly selected from HV, BPD, LD-SCLC, and ED-SCLC) in spotting buffer to a concentration of 1 mg mL^−1^ on the surface of an epoxy slide according to our previous publication.^26^ Each serum sample was spotted in triplicate, and the layout of the serum microarrays is shown in Fig. 4A. The three lectins (BS-I, UEA-I and GSL-I) that revealed significant differences (*p* < 0.05) in the serum glycopatterns according to the results of the respective lectin microarrays were selected to validate the differential expression levels of the targeted glycan structures in the individual serum (Fig. 4B). BS-I staining showed increased fluorescence intensities (FIs) (*p* ≤ 0.002) in the patients with LD-SCLC and ED-SCLC compared with individual HVs. UEA-I staining showed increased FIs in the individuals with LD-SCLC and ED-SCLC compared with the individuals with HVs (*p* ≤ 0.045). GSL-I staining showed decreased FIs in the individuals with ED-SCLC compared with the individuals with HVs (*p* < 0.001). These results are generally consistent with the results from the lectin microarrays.

**Fig. 5:**
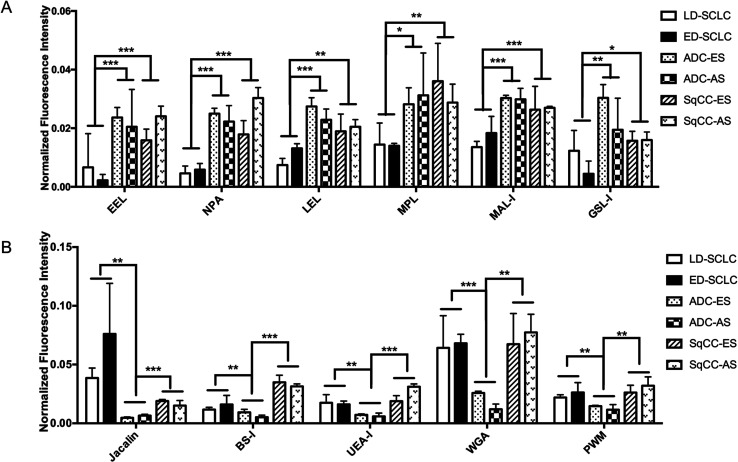
Systematic comparison of the glycoprotein glycopatterns between SCLC, ADC and SqCC sera with the lectin microarray. (A) Six lectins showed significant differences between the SCLC and NSCLC patients (all fold change ≥ 1.5, *p* < 0.05). (B) Five lectins showed significant differences between ADC and SCLC, SqCC patients (all fold change ≤ 0.67, *p* < 0.05).

Another serum microarray was fabricated to validate the differential expression levels of serum glycopatterns among HV, BPD, ADC and SqCC by spotting 120 individual serum samples (20 male and 10 female samples randomly selected from HV, BPD, ADC and SqCC). Each serum sample was repeatedly spotted three times, and the layout of the serum microarray is shown in Fig. 5A. Based on the results of the previous lectin microarrays, three different lectins (*p* < 0.05) (PTL-II, UEA-I and DBA) were selected for serum glycosylation changes in lung cancer, and further verified by the serum microarray to confirm if there were differences in the expression level of the target glycan structure in the individual serum samples. As a result, PTL-II had increased FIs in ADC-ES and ADC-AS compared with the HV group (*p* < 0.05). Moreover, it had a significant increase in alteration in SqCC-ES and SqCC-AS compared with the HV group. UEA-I had decreased FIs in ADC-AS compared with the HV group (*p* < 0.05). DBA had increased FIs in SqCC-AS compared with the HV group (*p* = 0.001) (Fig. 5B). These results are generally consistent with the results from the lectin microarrays.

### 3.5 Systematic comparison of the serum protein glycopatterns among SCLC, ADC and SqCC

There were 16 lectins (*e.g.*, RCA120, BS-I, and UEA-I), 24 lectins (*e.g.*, HHL, PTL-I, and MAL-II), and 18 lectins (*e.g.*, GSL-I, LEL, and ACA) that revealed significant differences in the serum protein glycopatterns from the patients with SCLC, ADC and SqCC compared with the controls (HV and HPD), respectively. A systematic comparative analysis of the serum protein glycopatterns among SCLC, ADC and SqCC was performed based on the above data, which showed that there were 6 lectins (EEL, NPA, LEL, MPL, MAL-I, and GSL-I) that exhibited significantly increased NFIs in ADC and SqCC compared with SCLC (all fold change ≥ 1.5, *p* ≤ 0.01), and almost an even level in NFIs (fold change range from 0.67 to 1.50, no significant difference) between ADC and SqCC (Fig. 5A). However, there were 5 lectins (Jacalin, BS-I, UEA-I, WGA, and PWM) that exhibited significantly decreased NFIs in ADC compared with SCLC and SqCC (all fold change ≥ 1.5, *p* ≤ 0.01), and Jacalin showed significantly increased NFIs in SCLC compared with SqCC-ES (all fold change ≥ 1.54, *p* ≤ 0.54), and BS-I exhibited significantly decreased NFIs in SCLC compared with SqCC (all fold change ≥ 1.5, *p* ≤ 0.01).

## 4 Discussion

Glycosylation is one of the most common post-translational modifications (PTMs) of secreted proteins and plays a critical role in signal recognition and conduction, cell–cell interaction, cell adhesion, malignant transformation and metastasis in the development of many cancers.^35^ In the diagnosis of diseases such as lung cancer, gastric cancer, breast cancer, and hepatocellular carcinoma, serum glycobiomarker identification has led to big breakthroughs and improvements in their detection, and thus has become a topic of increasing interest because of its high sensitivity and specificity.^36–39^ Moreover, the growing practice of glycobiology provides great promise in the discovery of glycosylated biomarkers for various diseases. As high-throughput glycan technology, lectin microarrays can be used for the rapid and accurate detection of multiple different carbohydrate structures without the need to release N- or O-linked glycans before analysis. Consequently, real information can be obtained about protein glycosylation.^28,40^

In the present study, 333 serum samples were collected for screening candidate lectins and constructing diagnostic models in retrospective cohort. SCLC with a high rate of metastasis and recurrence, is more aggressive and malignant than several types of lung cancer. More than 70% of patients are diagnosed with metastatic disease. Therefore, there is an urgent need for effective SCLC biomarkers to improve its diagnosis and achieve early detection. There were 16 lectin (*e.g.*, PHA-E + L, BS-I, and UEA-I) alterations in the serum glycopatterns between HV, BPD, LD-SCLC and ED-SCLC using lectin microarrays, serum microarrays, and statistical analysis. ADC is the most common type of lung cancer, which has obvious cellular and molecular characteristics, and its glands or ducts have a large amount of mucus. Because of its different pathological features and prognosis, ADC is classified as a type of NSCLC, which is totally different from SCLC. There were 24 lectin (*e.g.*, LEL, AAL, MAL-I, and PWM) alterations in the serum glycopatterns between HV, BPD, ADC-ES and ADC-AS using lectin microarrays, serum microarrays, and statistical analysis. SqCC is one form of NSCLC, which begins in the tissues that line the air passages in the lungs. It is also known as epidermoid carcinoma. Most SqCC of the lungs are located centrally, usually in the larger bronchi that join the trachea to the lung. There were 18 lectins (*e.g.*, MPL, BS-I, UEA-I) showing significant alterations in NFIs in the pooled serum among HV, BPD, SqCC-ES, and SqCC-AS.

The serum of patients with different cancers has its own specific glycoprotein glycans. When different types of cancer occur, the corresponding glycan type or expression level changes, and these changes can be quickly detected by the lectin microarray. In our previous studies, the results showed that in different cancers, the alterations in glycopatterns are totally different. In breast cancer, the glycans recognized by MAL-I, ECA, NPA, BPL, BS-I, PTL-II, DBA, PNA, PHA-E + L, UEA-I, and PWM showed differential expression.^41^ In gastric cancer, the glycans recognized by ECA, PSA, PHA-E + L, HHL, PNA, EEL, MPL, and GSL-I showed differential expression.^31^ In hepatic carcinoma, the glycans recognized by MAL-II, SNA, WFA, PNA, and AAL showed differential expression.^42^ In lung cancer, the alteration in the glycopattern is different from breast cancer, gastric cancer and hepatic carcinoma. Furthermore, different types lung cancer show different glycopatterns. The changes in the serum glycosylation levels are correlated with tumor pathological characteristics, and this correlation can be amplified by the lectin microarray signals, which provides a good reference for the rapid diagnosis of different diseases.

The fold changes in the serum glycopatterns based on the ratio of the NFIs of each lectin from the HV, BPD, SCLC, ADC, and SqCC patients are summarized in Table 2. We found that the blood group B antigen and high-mannose recognized by EEL and NPA increased in the patients with BPD, SCLC, ADC, and SqCC compared with HV, and SCLC, ADC, and SqCC compared with BPD. Thus, EEL and NPA have potential to become markers for the diagnosis of lung diseases. The biantennary complex-type *N*-glycan with outer Gal and Sia2-6Gal/GalNAc recognized by PHA-E + L and SNA decreased in the patients with SCLC compared with HV and BPD, but increased in the patients with SqCC-AS compared with HV and BPD. Therefore, SCLC and SqCC may be distinguished by PHA-E + L and SNA. Siaα2-3Galβ1-4Glc(NAc)/Glc recognized by MAL-II only reflected the change in ADC and core fucose recognized by AAL, and (GlcNAc)_*n*_ recognized by WGA, and BraBLDhed (LacNAc)_*n*_ recognized by PWM only reflected the change in SqCC. Thus, these lectins can be used to distinguish ADC and SqCC (Table 2). According to the research results, different combinations of lectins can be used to detect the type of lung cancer, and even its pathological stage.

**Table 2:** Fold change of the serum glycopatterns based on the ratio of the NFIs of each lectin from HVs, BPD, SCLC, ADC, and SqCC patients

Lectin	Compared with HV							Lectin	Compared with BPD					
	BPD	SCLC		ADC-ES/HV		SqCC			SCLC		ADC		SqCC	
	BPD/HV	LD/HV	ED/HV	ES/HV	AS/HV	ES/HV	AS/HV		LD/BPD	ED/BPD	ES/BPD	AS/BPD	ES/BPD	AS/BPD
EEL	6.707↑	5.940↑	1.994↑	14.210↑	21.478↑	21.144↑	18.316↑	EEL	—	0.297↑	2.119↑	3.202↑	3.153↑	2.731↑
NPA	4.655↑	1.743↑	2.218↑	6.751↑	11.439↑	9.438↑	8.404↑	NPA	0.374↑	0.476↑	—	2.457↑	2.027↑	1.805↑
MPL	—	—	—	3.193↑	2.541↑	2.495↑	2.764↑	MPL	—	—	2.619↑	2.084↑	2.046↑	2.267↑
LEL	—	—	—	1.776↑	1.915↑	2.593↑	2.158↑	LEL	0.501↑	—	—	—	1.836↑	1.527↑
GSL-I	2.371↑	—	0.504↓	1.775↑	1.794↑	3.421↑	2.194↑	GSL-I	0.585↑	0.213↑	—	—	—	—
DBA	1.870↑	—	—	1.938↑	2.829↑	2.376↑	2.815↑	DBA	0.588↓	—	—	1.513↑	—	1.505↑
LCA	0.532↓	—	0.614↓	0.480↓	0.231↓	0.237↓	0.201↓	LCA	1.840↓	—	—	0.434↑	0.445↑	0.378↑
BS-I	2.069↑	1.511↑	2.081↑	4.522↑	4.074↑	—	—	BS-I	—	—	2.185↑	1.969↑	0.586↓	0.329↓
UEA-I	1.914↑	1.754↑	1.625↑	1.901↑	3.143↑	—	0.597↓	UEA-I	—	—	—	1.642↑	0.380↑	0.312↑
PHA-E + L	—	0.649↓	0.660↓	—	—	—	—							
SNA	—	—	—	—	—	—	2.066↑	SNA	0.590↓	0.634↓	—	—	—	1.545↑
HHL	2.059↑	—	1.726↑	—	1.689↑	1.712↑	1.630↑	HHL	0.491↓	—	0.574↓	—	—	—
MAL-II	—	—	—	—	—	—	1.633↑	MAL-II	—	—	0.633↓	0.534↓	—	—
AAL	—	—	—	—	—	1.539↑	2.533↑	AAL	—	—	—	—	1.501↑	2.471↑
MAL-I	—	—	—	—	—	1.668↑	1.646↑	MAL-I	0.670↓	—	—	—	—	—
WGA	—	—	—	—	—	0.403↓	0.187↓	WGA	—	—	—	—	0.421↓	0.195↓
PWM	—	—	—	—	—	0.562↓	0.445↓	PWM	—	—	—	—	0.585↓	0.463↓

Untargeted abnormal growth of lung tissue often leads to a benign lung tumor, and benign lung tumors may be derived from many different structures of lung. Currently, very little is known about the causes of benign lung tumors and nodules. However, in general, benign lung tumors are usually caused by inflammation caused by infection, tuberculosis, lung abscess and non-infectious causes. Our results indicated that there were 8 lectins (*e.g.* BS-I, UEA-I, and RCA120) to show a significant increase in NFIs (fold change ≥ 1.87, *p* < 0.043) and 2 lectins (Jacalin and LCA) to show a significant decrease in NFIs (fold change ≤ 0.58, *p* < 0.043) in BPD compared with HV. Moreover, there were 6 lectins (*e.g.* BS-I, EEL, and HHL) to show a significant increase in NFIs (fold change ≥ 2.06, *p* < 0.043) in BPD compared with HV.

One limitation for this study is that our investigation does not refer to the mechanisms that cause the alterations in serum protein glycosylation in patients with LC. Past research revealed that fucosylation of E-cadherin activated the downstream gene to be translated, which promoted the dimerization and phosphorylation of EGFR, and this can ultimately lead to lung cancer cell proliferation and metastasis.^43^ Thus, it is necessary to deeply study the mechanism of glycosylation and the development of lung cancer. The second limitation was the lack of enough clinical samples from patients of LCC or ASC. The sample size was statistically non-significant. Therefore, this study aimed to study three common types of lung cancer. Another concern is the lack of verification about the different glycopatterns among SCLC, ADC and SqCC. These findings were calculated based on previous research results and are of great significance for the construction of a lung cancer diagnostic model. Therefore, we have collected more clinical samples for further research, with the aim of identifying the lung cancer types and stages more accurately. Studies in larger cohorts enrolling more patients with the different subtypes also need to be carried out in the future.

In conclusion, we systematically investigated the correlation between the alterations in serum glycosylation related to three main types of lung cancer (SCLC, ADC and SqCC) using lectin microarrays, which helped us gain new insight into the serum protein glycopatterns. Furthermore, the different or similar alterations of glycoprotein glycopatterns among SCLC, ADC and SqCC were systematically compared. There were 16 lectins (*e.g.*, RCA120, BS-I, and UEA-I), 24 lectins (*e.g.*, HHL, PTL-I, and MAL-II), and 18 lectins (*e.g.*, GSL-I, LEL, and ACA) that revealed significant differences in serum protein glycopatterns from the patients with SCLC, ADC and SqCC compared with the controls (HV and HPD), respectively. There were 6 lectins (*e.g.*, EEL, NPA, and LEL) that exhibited significantly increased NFIs in ADC and SqCC compared with SCLC. Also, there were 5 lectins (*e.g.*, Jacalin, BS-I, and UEA-I) that exhibited significantly decreased NFIs in ADC compared with SCLC and SqCC. This study can facilitate the discovery of potential biomarkers for the differential diagnosis of lung cancer based on the precise alterations in serum protein glycopatterns.

## Abbreviations

SCLC: Small-cell lung cancer
NSCLC: Non-small-cell carcinoma
ADC: Lung adenocarcinoma
SqCC: Squamous cell lung cancer
HV: Healthy volunteer
BPD: Benign pulmonary disease
LD-SCLC: Limited disease small cell lung cancer
ED-SCLC: Extensive disease small cell lung cancer
ADC-ES: Lung adenocarcinoma early stage
ADC-AS: Lung adenocarcinoma advanced stage
SqCC-ES: Squamous cell lung cancer early stage
SqCC-AS: Squamous cell lung cancer advanced stage

## Author contributions

All authors participated in literature research and data classification. LJ, TM, XL and YZ: design of study and manuscript preparation; YL, HY and MC: serum collection; HD, JS and ZZ: data statistics; ZL: critical revision of the manuscript.

## Conflicts of interest

The authors declare that the research was conducted in the absence of any commercial or financial relationships that could be construed as a potential conflict of interest.

## Supplementary Material

RA-010-C9RA10077F-s001

## References

[cit1] Siegel R., Ma J., Zou Z., Jemal A. (2014). Ca-Cancer J. Clin..

[cit2] Siegel R. L., Miller K. D., Jemal A. (2015). Ca-Cancer J. Clin..

[cit3] Torre L. A., Bray F., Siegel R. L., Ferlay J., Lortet-Tieulent J., Jemal A. (2015). Ca-Cancer J. Clin..

[cit4] Bray F., Ferlay J., Soerjomataram I., Siegel R. L., Torre L. A., Jemal A. (2018). Ca-Cancer J. Clin..

[cit5] Chen W., Zheng R., Baade P. D., Zhang S., Zeng H., Bray F., Jemal A., Yu X. Q., He J. (2016). Ca-Cancer J. Clin..

[cit6] Torre L. A., Siegel R. L., Jemal A. (2016). Adv. Exp. Med. Biol..

[cit7] Addario B. J. (2015). Ann. Transl. Med..

[cit8] Siegel R., Naishadham D., Jemal A. (2013). Ca-Cancer J. Clin..

[cit9] Cheng J. H., Liu W. S., Li Z. M., Wang Z. G. (2007). Chin. J. Integr. Med..

[cit10] World Health Organization , http://who.int/gho/database/en/, accessed June 21, 2018, 10.1111/his.12157

[cit11] Li J. J., Li R., Wang W., Zhang B., Song X., Zhang C., Gao Y., Liao Q., He Y., You S., Tan Z., Luo X., Li Y., Tang M., Weng X., Yi W., Peng S., Liu S., Tan Y., Bode A. M., Cao Y. (2018). Mol. Oncol..

[cit12] Shinkai T., Saijo N., Tominaga K., Eguchi K., Shimizu E., Sasaki Y., Fujita J., Futami H., Ohkura H., Suemasu K. (1986). Cancer.

[cit13] Pujol J. L., Grenier J., Daures J. P., Daver A., Pujol H., Michel F. B. (1993). Cancer Res..

[cit14] Song X., Han X., Yu F., Zhang X., Chen L., Lv C. (2018). Theranostics.

[cit15] Zhang X., He N., Huang Y., Yu F., Li B., Lv C., Chen L. (2019). Sens. Actuators, B.

[cit16] Stowell S. R., Ju T. Z., Cummings R. D. (2015). Annu. Rev. Pathol.: Mech. Dis..

[cit17] Mehta A., Herrera H., Block T. (2015). Adv. Cancer Res..

[cit18] Ueda K., Fukase Y., Katagiri T., Ishikawa N., Irie S., Sato T. A., Ito H., Nakayama H., Miyagi Y., Tsuchiya E., Kohno N., Shiwa M., Nakamura Y., Daigo Y. (2009). Proteomics.

[cit19] Vasseur J. A., Goetz J. A., Alley W. R., Novotny M. V. (2012). Glycobiology.

[cit20] Kannagi R., Fukushi Y., Tachikawa T., Noda A., Shin S., Shigeta K., Hiraiwa N., Fukuda Y., Inamoto T., Hakomori S. (1986). et al.. Cancer Res..

[cit21] Ruhaak L. R., Taylor S. L., Stroble C., Nguyen U. T., Parker E. A., Song T., Lebrilla C. B., Rom W. N., Pass H., Kim K., Kelly K., Miyamoto S. (2015). J. Proteome Res..

[cit22] Zhang D., Li X., Liu X., Wang Y., Zhang M., Wang Q., Chen T., Li Z. (2020). Proteomics: Clin. Appl..

[cit23] Chen T., He C., Zhang M., Li X., Liu X., Liu Y., Zhang D., Li Z. (2019). J. Cancer.

[cit24] Widlak P., Pietrowska M., Polanska J., Marczyk M., Ros-Mazurczyk M., Dziadziuszko R., Jassem J., Rzyman W. (2016). Lung Cancer.

[cit25] Zeng X. M., Hood B. L., Sun M., Conrads T. P., Day R. S., Weissfeld J. L., Siegfried J. M., Bigbee W. L. (2010). J. Proteome Res..

[cit26] Hirabayashi J., Yamada M., Kuno A., Tateno H. (2013). Chem. Soc. Rev..

[cit27] Liang Y., Ma T., Thakur A., Yu H., Gao L., Shi P., Li X., Ren H., Jia L., Zhang S., Li Z., Chen M. (2015). Glycobiology.

[cit28] Gupta G., Surolia A., Sampathkumar S. G. (2010). OMICS.

[cit29] Zhong Y., Qin Y., Dang L., Jia L., Zhang Z., Wu H., Cui J., Bian H., Li Z. (2015). Proteomics.

[cit30] Qin Y., Zhong Y., Zhu M., Dang L., Yu H., Chen Z., Chen W., Wang X., Zhang H., Li Z. (2013). J. Proteome Res..

[cit31] Shu J., Yu H., Li X., Zhang D., Liu X., Du H., Zhang J., Yang Z., Xie H., Li Z. (2017). Oncotarget.

[cit32] Zhong Y., Zhang J., Yu H., Zhang J., Sun X. X., Chen W., Bian H., Li Z. (2015). Biochem. Biophys. Res. Commun..

[cit33] Zhu H., Liu M., Yu H., Liu X., Zhong Y., Shu J., Fu X., Cai G., Chen X., Geng W., Yang X., Wu M., Li Z., Zhang D. (2017). J. Diabetes Res..

[cit34] Badr H. A., Alsadek D. M., Darwish A. A., Elsayed A. I., Bekmanov B. O., Khussainova E. M., Zhang X., Cho W. C., Djansugurova L. B., Li C. Z. (2014). Expert Rev. Proteomics.

[cit35] Nie H., Liu X., Zhang Y., Li T., Zhan C., Huo W., He A., Yao Y., Jin Y., Qu Y., Sun X. L., Li Y. (2015). Sci. Rep..

[cit36] Veillon L., Fakih C., Abou-El-Hassan H., Kobeissy F., Mechref Y. (2018). ACS Chem. Neurosci..

[cit37] Oliveira-Ferrer L., Legler K., Milde-Langosch K. (2017). Semin. Cancer Biol..

[cit38] Vecchio G., Parascandolo A., Allocca C., Ugolini C., Basolo F., Moracci M., Strazzulli A., Cobucci-Ponzano B., Laukkanen M. O., Castellone M. D., Tsuchida N. (2017). Oncotarget.

[cit39] Leng Q., Lin Y., Zhan M., Jiang F. (2018). Oncotarget.

[cit40] Shi Y. Q., He Q., Zhao Y. J., Wang E. H., Wu G. P. (2013). Cytotechnology.

[cit41] Liu X. W., Yu H. J., Qiao Y., Yang J. J., Shu J., Zhang J. X., Zhang Z. W., He J. J., Li Z. (2018). Ebiomedicine.

[cit42] Qin Y., Zhong Y., Ma T., Wu F., Wu H., Yu H., Huang C., Li Z. (2016). Glycoconjugate J..

[cit43] Jia L., Zhang J., Ma T., Guo Y., Yu Y., Cui J. (2018). Front. Oncol..

