# Supplementary material for: Alterations in serum protein glycopatterns related to small cell lung cancer, adenocarcinoma and squamous carcinoma of the lung
Source: RSC Adv. 2020 Feb 18;10(12):7181–93. doi: 10.1039/c9ra10077f (PMC9049724; doi:10.1039/c9ra10077f)
Supplement: RA-010-C9RA10077F-s001 [file RA-010-C9RA10077F-s001.pdf]

Electronic Supplementary Material (ESI) for RSC Advances.
This journal is © The Royal Society of Chemistry 2020

Alterations of Serum Protein Glycopatterns related to Lung Cancer (SCLC, ADC, and SqCC)

Liyuan Jia[1†], Tianran Ma[1†], Yiqian Liang[2], Haoqi Du[1], Jian Shu[1], Xiawei Liu[1], Zhiwei Zhang[1], Hanjie Yu[1], Mingwei Chen[2*], Zheng Li[1*]

Supporting Table 1. Glycopattern in pooled serum from HVs and patients with benign lung disease and LD-SCLC, ED-SCLC by the lectin microarray analysis based on data of 16 lectins giving significant signal.

**Lectin**	**Specificity**	**HV**	**BPD**	**LD-SCLC**	**ED-SCLC**
Jacalin	Galβ1-3GalNAcα-Ser/Thr(T), GalNAcα-Ser/Thr(Tn)	0.069±0.016	0.04±0.002	0.039±0.008	0.076±0.043
HHL	High-Mannose, Manα1-3Man, Manα1-6Man, Man5-GlcNAc2-Asn	0.008±0.001	0.016±0.005	0.008±0.002	0.013±0.002
WFA	terminating in GalNAcα/β1-3/6Gal	0.016±0.003	0.021±0.007	0.027±0.004	0.023±0.004
PHA-E	Bisecting GlcNAc, biantennary complex-type N-glycan with outer Gal	0.027±0.004	0.034±0.005	0.023±0.004	0.019±0.004
EEL	Galα1-3(Fucα1-2) Gal (blood group B antigen)	0.001±0.002	0.008±0.012	0.007±0.012	0.002±0.002
LTL	Fucα1-2Galβ1-4GlcNAc, Fucα1-3(Galβ1-4) GlcNAc	0.021±0.003	0.024±0.007	0.015±0.001	0.021±0.007
LEL	(GlcNAc)n, high mannose-type N-glycans	0.011±0.001	0.015±0.006	0.007±0.002	0.013±0.002
GSL-I	αGalNAc, αGal, anti-A and B	0.009±0.01	0.021±0.003	0.012±0.007	0.004±0.004
DBA	αGalNAc, Tn antigen, GalNAcα1-3(Fucα1-2) Gal (blood group A antigen)	0.009±0.001	0.016±0.006	0.009±0.002	0.012±0.004
LCA	α-D-Man, Fucα-1,6GlcNAc, α-D-Glc	0.187±0.013	0.099±0.037	0.183±0.028	0.114±0.082
STL	trimers and tetramers of GlcNAc, core (GlcNAc) of N-glycan, oligosaccharide containing GlcNAc and MurNAc	0.033±0.007	0.026±0.012	0.052±0.005	0.044±0.004
BS-I	α-Gal, α-GalNAc, Galα-1,3Gal, Galα-1,6Glc	0.008±0.006	0.016±0.004	0.012±0.002	0.016±0.008
NPA	High-Mannose, Manα1-6Man	0.003±0.004	0.012±0.007	0.005±0.002	0.006±0.002

UEA-I	Fucα1-2Galβ1-4Glc(NAc)	0.01 ±0.009	0.019 ±0.001	0.017 ±0.007	0.016 ±0.003
RCA120	β-Gal, Galβ-1,4GlcNAc (type II), Galβ1-3GlcNAc (type I)	0 ±0	0.014 ±0.005	0.018 ±0.003	0.021 ±0.003
SNA	Sia2-6Gal/GalNAc	0.025 ±0.005	0.033 ±0.009	0.019 ±0.005	0.021 ±0.005

Supporting Table 2. Fold change of the serum glycopatterns based upon ratio of the NFIs of each lectin from HVs, BPD, LD-SCLC and ED-SCLC patients.

**Lectin**	**Specificity**	**Compared with HV (*p* value)** [a]			**Compared with each other (*p* value)** [b]		
		**BPD/HV**	**LD-SCLC/HV**	**ED-SCLC/HV**	**LD-SCLC/BPD**	**ED-SCLC/BPD**	**ED-SCLC/LD-SCLC**
BS-I	α-Gal, α-GalNAc, Galα-1,3Gal, Galα-1,6Glc	2.07(0.008)	1.51(0.024)	2.08(0.002)	-	-	-
RCA120	β-Gal, Galβ-1,4GlcNAc (type II), Galβ1-3GlcNAc (type I)	/ (<0.001)	/ (<0.001)	/ (<0.001)	-	-	-
UEA-I	Fucα1-2Galβ1-4Glc(NAc)	1.91(0.017)	-	1.62(0.026)	-	-	-
RCA120	β-Gal, Galβ-1,4GlcNAc (type II), Galβ1-3GlcNAc (type I)	/ (<0.001)	/ (<0.001)	/ (<0.001)	-	-	-
WFA	terminating in GalNAcα/β1-3/6Gal	-	1.69(<0.001)	-	-	-	-
SNA	Sia2-6Gal/GalNAc	-	-	-	0.59(0.005)	0.63(0.049)	-
LEL	(GlcNAc)n, high mannose-type N-glycans	-	-	-	0.50(0.021)	-	1.76(0.014)
LTL	Fucα1-2Galβ1-4GlcNAc, Fucα1-3(Galβ1-4) GlcNAc	-	-	-	0.60(0.006)	-	-
PHA-E	Bisecting GlcNAc, biantennary complex-type N-glycan with outer Gal	-	-	-	-	0.55(0.016)	-
Jacalin	Galβ1-3GalNAcα-Ser/Thr(T), GalNAcα-Ser/Thr(Tn)	0.58(0.043)	0.56(0.006)	-	-	1.91(0.023)	1.97(0.042)
LCA	α-D-Man, Fucα-1,6GlcNAc, α-D-Glc	0.53(0.021)	-	0.61(<0.001)	1.84(0.032)	-	0.63(0.028)
EEL	Galα1-3(Fucα1-2) Gal (blood group B antigen)	6.71(0.006)	5.94(<0.001)	1.99(0.023)	-	0.30(0.047)	0.34(0.012)

GSL-I	αGalNAc, αGal, anti-A and B	2.37(0.043)	-	0.50(0.016)	0.58(0.035)	0.21(0.039)	0.36(<0.001)
NPA	High-Mannose, Manα1-6Man	4.66(<0.001)	-	2.22(0.041)	0.37(0.005)	0.48(0.038)	-
HHL	High-Mannose, Manα1-3Man, Manα1-6Man, Man5-GlcNAc2-Asn	2.06(0.029)	-	1.73(0.016)	0.49(0.030)	-	1.71(0.004)
STL	trimers and tetramers of GlcNAc, core (GlcNAc) of N-glycan, oligosaccharide containing GlcNAc and MurNAc	-	1.55(0.046)	-	1.97(0.007)	1.68(0.019)	-
DBA	αGalNAc, Tn antigen, GalNAcα1-3(Fucα1-2) Gal (blood group A antigen)	1.87(0.010)	-	-	0.59(0.006)	-	-

[a] The NFIs of each lectin from BLD, LD-SCLC, and ED-SCLC groups were compared with that from HV group based on their fold change (i.e., BLD/HV, LD-SCLC/HV, and ED-SCLC/HV), respectively.

[b]The NFIs of each lectin from three disease groups were compared with BPD and each other based upon fold change in pairs, i.e., LD-SCLC/BLD, ED-SCLC/BLD, and ED-SCLC/LD-SCLC. -, no significant difference; /, the denominator of the fold-change was zero.

Supporting Table 3. Glycopattern in pooled serum from HVs, BPD, ADC-ES and ADC-AS patients by the lectin microarray analysis based on data of 23 lectins giving significant signal.

Lectin	Specificity	HV	BPD	ES-ADC	AS-ADC
HHL	High-Mannose, Manα1-3Man, Manα1-6Man, Man5-GlcNAc2-Asn	0.008±0.001	0.016±0.005	0.013±0.001	0.013±0.01
WFA	terminating in GalNAcα/β1-3/6Gal	0.016±0.003	0.021±0.007	0.024±0.005	0.035±0.003
MAL-II	Siaα2-3Galβ1-4Glc(NAc)/Glc	0.021±0.004	0.029±0.005	0.03±0.001	0.034±0.001
PTL-I	GalNAc, GalNAcα-1,3Gal, GalNAcα-1,3Galβ-1,3/4Glc	0.014±0.003	0.018±0.003	0.021±0.003	0.017±0.008
EEL	Galα1-3(Fucα1-2) Gal (blood group B antigen)	0.001±0.002	0.008±0.012	0.024±0.003	0.021±0.013
AAL	Fucα1-6 GlcNAc (core fucose), Fucα1-3(Galβ1-4) GlcNAc	0.023±0.005	0.024±0	0.036±0.004	0.06±0.018
MPL	Galβ1-3GalNAc, GalNAc	0.011±0.009	0.014±0.008	0.028±0.006	0.031±0.015
LEL	(GlcNAc)n, high mannose-type N-glycans	0.011±0.001	0.015±0.006	0.027±0.003	0.023±0.004
GSL-I	αGalNAc, αGal, anti-A and B	0.009±0.01	0.021±0.003	0.031±0.005	0.02±0.011
DBA	αGalNAc, Tn antigen, GalNAcα1-3(Fucα1-2) Gal (blood group A antigen)	0.009±0.001	0.016±0.006	0.021±0.005	0.024±0.001
LCA	α-D-Man, Fucα-1,6GlcNAc, α-D-Glc	0.187±0.013	0.099±0.037	0.044±0.008	0.038±0.014

RCA120	β-Gal, Galβ-1,4GlcNAc (type II), Galβ1-3GlcNAc (type I)	0 ±0	0.014 ±0.005	0.018 ±0.003	0.021 ±0.003
STL	trimers and tetramers of GlcNAc, core (GlcNAc) of N-glycan, oligosaccharide containing GlcNAc and MurNAc	0.033 ±0.007	0.026 ±0.012	0.027 ±0.002	0.042 ±0.008
BS-I	α-Gal, α-GalNAc, Galα-1,3Gal, Galα-1,6Glc	0.008 ±0.006	0.016 ±0.004	0.009 ±0.003	0.005 ±0.002
ConA	High-Mannose, Manα1-6(Manα1-3) Man, terminal GlcNAc	0.021 ±0.004	0.021 ±0.003	0.034 ±0.001	0.028 ±0.008
PTL-II	Gal, blood group H, T-antigen	0.01 ±0.002	0.013 ±0.003	0.024 ±0.008	0.014 ±0.003
VVA	terminal GalNAc, GalNAcα-Ser/Thr(Tn), GalNAcα1-3Gal	0.029 ±0.006	0.034 ±0.009	0.041 ±0.002	0.026 ±0.003
NPA	High-Mannose, Manα1-6Man	0.003 ±0.004	0.012 ±0.007	0.025 ±0.002	0.022 ±0.006
WGA	Multivalent Sia and $(GlcNAc)_n$	0.064 ±0.012	0.061 ±0.021	0.026 ±0.002	0.012 ±0.004
UEA-I	Fucα1-2Galβ1-4Glc(NAc)	0.01 ±0.009	0.019 ±0.001	0.007 ±0.001	0.006 ±0.003
PWM	BraBLDhed (LacNAc)n	0.026 ±0.003	0.025 ±0.001	0.015 ±0.001	0.012 ±0.004
MAL-I	Galβ-1,4GlcNAc, Siaα2-3Gal, Galβ1-3GlcNAc, Siaα2-3	0.018 ±0.002	0.02 ±0.006	0.03 ±0.001	0.03 ±0.004
SNA	Sia2-6Gal/GalNAc	0.025 ±0.005	0.033 ±0.009	0.034 ±0.001	0.051 ±0.015

Supporting Table 4. Fold change of the serumry glycopatterns based upon ratio of the NFIs of each lectin from HVs, BPD, ADC-ES and ADC-AS patients.

Lectin	Specificity	Compared with HV (*p* value) [a]			Compared with each other (*p* value) [b]		
		**BPD/HV**	**ADC-ES/HV**	**ADC-AS/HV**	**ADC-ES /BPD**	**ADC-AS /BPD**	**ADC-AS/ADC-ES**
HHL	High-Mannose, Manα1-3Man, Manα1-6Man, Man5-GlcNAc2-Asn	2.06(0.024)	1.72(0.008)	1.64(0.041)	-	-	-
PTL-I	GalNAc, GalNAcα-1,3Gal, GalNAcα-1,3Galβ-1,3/4Glc	-	1.55(0.016)	-	-	-	-
MAL-II	Siaα2-3Galβ1-4Glc(NAc)/Glc	-	-	1.64(0.001)	-	-	-
STL	trimers and tetramers of GlcNAc, core (GlcNAc) of N-glycan, oligosaccharide containing GlcNAc and MurNAc	-	-	-	-	1.60(0.017)	1.57(0.009)
VVA	terminal GalNAc, GalNAcα-Ser/Thr(Tn), GalNAcα1-3Gal	-	-	-	-	-	0.64(0.011)
Jacalin	Galβ1-3GalNAcα-Ser/Thr(T), GalNAcα-Ser/Thr(Tn)	0.58(0.043)	0.07(0.016)	0.09(0.005)	0.12(0.032)	0.16(0.007)	-
LCA	α-D-Man, Fucα-1,6GlcNAc, α-D-Glc	0.53(0.014)	0.24(0.001)	0.20(0.001)	0.45(0.033)	0.38(0.025)	-

WGA	Multivalent Sia and (GlcNAc)n	-	0.40(0.005)	0.19(0.001)	0.42(0.008)	0.20(0.001)	0.46(0.020)
PWM	BraBPDhed (LacNAc)n	-	0.56(0.001)	0.45(<0.001)	0.59(0.002)	0.20(<0.001)	-
BS-I	α-Gal, α-GalNAc, Galα-1,3Gal, Galα-1,6Glc	2.07(0.043)	-	-	0.59(0.012)	0.33(0.030)	0.56(0.038)
UEA-I	Fucα1-2Galβ1-4Glc(NAc)	1.91(0.045)	-	0.60(0.012)	0.38(0.020)	0.20(0.023)	-
EEL	Galα1-3(Fucα1-2) Gal (blood group B antigen)	6.71(0.041)	21.24(0.016)	18.44(0.037)	3.17(0.032)	2.75(0.011)	-
NPA	High-Mannose, Manα1-6Man	4.66(0.040)	9.48(<0.001)	8.46(0.001)	2.04(0.013)	1.82(0.037)	-
DBA	αGalNAc, Tn antigen, GalNAcα1-3(Fucα1-2) Gal (blood group A antigen)	1.87(0.046)	2.39(0.006)	2.83(0.001)	-	1.52(0.033)	-
RCA120	β-Gal, Galβ-1,4GlcNAc (type II), Galβ1-3GlcNAc (type I)	/ (<0.001)	/ (<0.001)	/ (<0.001)	-	1.53(<0.001)	-
GSL-I	αGalNAc, αGal, anti-A and B	2.37(0.027)	3.44(0.019)	2.21(0.049)	-	-	0.64(0.022)
AAL	Fucα1-6 GlcNAc (core fucose), Fucα1-3(Galβ1-4) GlcNAc	-	1.55(0.014)	2.55(0.002)	1.51(0.015)	2.49(0.018)	1.65(0.018)
MPL	Galβ1-3GalNAc, GalNAc	-	2.51(0.041)	2.78(0.024)	2.06(0.031)	2.28(0.041)	-

LEL	(GlcNAc)n, high mannose-type N-glycans	-	2.60(<0.001)	2.17(0.003)	1.84(0.003)	1.54(0.040)	-
MAL-I	Galβ-1,4GlcNAc, Siaα2-3Gal, Galβ1-3GlcNAc, Siaα2-3	-	1.68(0.004)	1.66(0.005)	1.50(0.012)	0.20(0.014)	-
WFA	terminating in GalNAcα/β1-3/6Gal	-	1.51(0.018)	2.15(0.001)	-	1.68(0.007)	-
ConA	High-Mannose, Manα1-6(Manα1-3) Man, terminal GlcNAc	-	1.57(0.013)	-	1.60(0.031)	-	-
PTL-II	Gal, blood group H, T-antigen	-	2.33(0.010)	-	1.78(0.032)	-	0.61(0.010)
SNA	Sia2-6Gal/GalNAc	-	-	2.08(0.008)	-	0.20(0.044)	1.51(0.015)

[a] [a] The NFIs of each lectin from HB, BPD, ADC-ES and ADC-AS groups were compared with that from HV group based on their fold change (i.e., BPD/HB, ADC-ES/HV, and ADC-AS/HV), respectively.

[b]The NFIs of each lectin from three disease groups were compared with that from BPD group based on their fold change and compared with each other on their fold change (i.e., ADC-ES/BPD, ADC-AS/BPD, and ADC-AS/ADC-ES. -, no significant difference; /, the denominator of the fold change was zero).

Supporting Table 5. Glycopatternin in sera from HVs, BPD, SqCC-ES and SqCC-AS by the lectin microarray analysis based on data of 19 lectins giving significant signal.

Lectin	Specificity	HV	BPD	SqCC-ES	SqCC-AS
HHL	High-Mannose, Manα1-3Man, Manα1-6Man, Man5-GlcNAc2-Asn	0.008±0.001	0.016±0.005	0.009±0.009	0.013±0.004
MAL-II	Siaα2-3Galβ1-4Glc(NAc)/Glc	0.021±0.004	0.029±0.005	0.018±0.007	0.016±0.004
PHA-E	Bisecting GlcNAc, biantennary complex-type N-glycan with outer Gal	0.027±0.004	0.034±0.005	0.03±0.005	0.022±0.004
SJA	Terminal in GalNAc and Gal	0.043±0.008	0.039±0.001	0.032±0.005	0.022±0.003
EEL	Galα1-3(Fucα1-2) Gal (blood group B antigen)	0.001±0.002	0.008±0.012	0.016±0.004	0.024±0.004
MPL	Galβ1-3GalNAc, GalNAc	0.011±0.009	0.014±0.008	0.037±0.013	0.029±0.006
LEL	(GlcNAc)n, high mannose-type N-glycans	0.011±0.001	0.015±0.006	0.019±0.006	0.02±0.002
GSL-I	αGalNAc, αGal, anti-A and B	0.009±0.01	0.021±0.003	0.016±0.003	0.016±0.003
DBA	αGalNAc, Tn antigen, GalNAcα1-3(Fucα1-2) Gal (blood group A antigen)	0.009±0.001	0.016±0.006	0.017±0.007	0.025±0.003
LCA	α-D-Man, Fucα-1,6GlcNAc, α-D-Glc	0.187±0.013	0.099±0.037	0.09±0.053	0.044±0.012
RCA120	β-Gal, Galβ-1,4GlcNAc (type II), Galβ1-3GlcNAc (type I)	0±0	0.014±0.005	0.028±0.002	0.021±0.002
STL	trimers and tetramers of GlcNAc, core (GlcNAc) of N-glycan, oligosaccharide containing GlcNAc and MurNAc	0.033±0.007	0.026±0.012	0.049±0.012	0.04±0.007

BS-I	α-Gal, α-GalNAc, Galα-1,3Gal, Galα-1,6Glc	0.008±0.006	0.016±0.004	0.035±0.007	0.032±0.002
PTL-II	Gal, blood group H, T-antigen	0.01±0.002	0.013±0.003	0.02±0.008	0.026±0.003
NPA	High-Mannose, Manα1-6Man	0.003±0.004	0.012±0.007	0.018±0.005	0.031±0.004
PSA	α-D-Man, Fucα-1,6GlcNAc, α-D-Glc	0.037±0.009	0.029±0.005	0.027±0.004	0.021±0.002
ACA	Galβ1-3GalNAcα-Ser/Thr (T)	0.028±0.002	0.032±0.002	0.038±0.013	0.046±0.004
UEA-I	Fucα1-2Galβ1-4Glc(NAc)	0.01±0.009	0.019±0.001	0.019±0.005	0.032±0.002
BPL	Galβ1-3GalNAc, Terminal GalNAc	0.033±0.007	0.031±0.001	0.018±0.008	0.032±0.004

Supporting Table 6. Fold change of the serum glycopatterns based upon ratio of the NFIs of each lectin from HVs, BPD, SqCC-ES and SqCC-AS patients.

Lectin	Specificity	Compared with HV (*p* value) [a]			Compared with each other (*p* value) [b]		
		**BPD/HV**	**SqCC-ES/HV**	**SqCC-AS/HV**	**SqCC-ES/BPD**	**SqCC-AS/BPD**	**SqCC-AS/SqCC-ES**
GSL-I	αGalNAc, αGal, anti-A and B	2.37(0.025)	1.80(0.001)	1.82(0.037)	-	-	-
LEL	(GlcNAc)n, high mannose-type N-glycans	-	1.80(<0.001)	1.94(0.003)	-	-	-
ACA	Galβ1-3GalNAcα-Ser/Thr (T)	-	-	1.68(0.004)	-	-	-
PSA	α-D-Man, Fucα-1,6GlcNAc, α-D-Glc	-	-	0.58(0.019)	-	-	-
STL	trimers and tetramers of GlcNAc, core (GlcNAc) of N-glycan, oligosaccharide containing GlcNAc and MurNAc	-	-	-	1.85(0.006)	1.51(0.002)	-
MAL-II	Siaα2-3Galβ1-4Glc(NAc)/Glc	-	-	-	0.64(0.011)	0.54(0.008)	-
Jacalin	Galβ1-3GalNAcα-Ser/Thr(T), GalNAcα-Ser/Thr(Tn)	0.58(0.043)	0.27(0.043)	0.22(0.002)	0.48(0.019)	0.38(0.026)	-
LCA	α-D-Man, Fucα-1,6GlcNAc, α-D-Glc	0.53(0.013)	0.48(0.008)	0.23(0.035)	-	0.44(0.008)	0.48(0.013)

BPL	Galβ1-3GalNAc, Terminal GalNAc	-	0.54(0.038)	-	0.58(0.015)	-	1.79(0.008)
SJA	Terminal in GalNAc and Gal	-	-	0.52(0.012)	-	0.58(0.003)	-
EEL	Galα1-3(Fucα1-2) Gal (blood group B antigen)	6.71(0.038)	14.34(0.003)	21.81(0.001)	2.14(<0.001)	3.25(<0.001)	1.52(0.0029)
BS-I	α-Gal, α-GalNAc, Galα-1,3Gal, Galα-1,6Glc	2.07(0.027)	4.58(<0.001)	4.14(0.002)	2.21(<0.001)	2.00(0.005)	-
NPA	High-Mannose, Manα1-6Man	4.66(0.042)	6.84(0.005)	11.62(0.020)	-	2.50(0.002)	1.70(0.015)
UEA-I	Fucα1-2Galβ1-4Glc(NAc)	1.91(0.047)	1.93(0.037)	3.19(0.001)	-	1.67(<0.001)	1.66(0.023)
RCA120	β-Gal, Galβ-1,4GlcNAc (type II), Galβ1-3GlcNAc (type I)	/ (<0.001)	/ (<0.001)	/ (<0.001)	2.02(<0.001)	-	-
DBA	αGalNAc, Tn antigen, GalNAcα1-3(Fucα1-2) Gal (blood group A antigen)	1.87(0.028)	1.97(0.046)	2.87(0.018)	-	1.54(0.006)	-
MPL	Galβ1-3GalNAc, GalNAc	-	3.23(0.012)	2.58(0.019)	2.65(0.015)	2.11(0.028)	-
PTL-II	Gal, blood group H, T-antigen	-	2.01(0.030)	2.59(0.010)	1.54(0.003)	1.99(0.010)	-
HHL	High-Mannose, Manα1-3Man, Manα1-6Man, Man5-GlcNAc2-Asn	2.06(0.017)	-	1.71(0.015)	0.58(0.015)	-	-

[a] The NFIs of each lectin from HB, BPD, SqCC-ES and SqCC-AS groups were compared with that from HV group based on their fold change (i.e., BPD/HB, SqCC-ES/HV, and SqCC-AS/HV), respectively.

[b]The NFIs of each lectin from three disease groups were compared with that from BPD group based on their fold change and compared with each other on their fold change (i.e., SqCC-ES/BPD, SqCC-AS/BPD, and SqCC-AS/SqCC-ES. -, no significant difference; /, the denominator of the fold change was zero).
